# Mechanism of Viral DNA Packaging in Phage T4 Using Single-Molecule Fluorescence Approaches

**DOI:** 10.3390/v16020192

**Published:** 2024-01-26

**Authors:** Souradip Dasgupta, Julie A. Thomas, Krishanu Ray

**Affiliations:** 1 Division of Vaccine Research, Institute of Human Virology, University of Maryland School of Medicine, 725 West Lombard Street, Baltimore, MD 21201, USA; 2 Thomas H. Gosnell School of Life Sciences, Rochester Institute of Technology, Rochester, NY 14623, USA; jatsbi@rit.edu; 3 Department of Biochemistry and Molecular Biology, University of Maryland School of Medicine, 725 West Lombard Street, Baltimore, MD 21201, USA

**Keywords:** T4 phage, DNA packaging, large terminase, small terminase, molecular motor, Single Molecule Fluorescence, FCS, FRET

## Abstract

In all tailed phages, the packaging of the double-stranded genome into the head by a terminase motor complex is an essential step in virion formation. Despite extensive research, there are still major gaps in the understanding of this highly dynamic process and the mechanisms responsible for DNA translocation. Over the last fifteen years, single-molecule fluorescence technologies have been applied to study viral nucleic acid packaging using the robust and flexible T4 in vitro packaging system in conjunction with genetic, biochemical, and structural analyses. In this review, we discuss the novel findings from these studies, including that the T4 genome was determined to be packaged as an elongated loop via the colocalization of dye-labeled DNA termini above the portal structure. Packaging efficiency of the TerL motor was shown to be inherently linked to substrate structure, with packaging stalling at DNA branches. The latter led to the design of multiple experiments whose results all support a proposed torsional compression translocation model to explain substrate packaging. Evidence of substrate compression was derived from FRET and/or smFRET measurements of stalled versus resolvase released dye-labeled Y-DNAs and other dye-labeled substrates relative to motor components. Additionally, active in vivo T4 TerS fluorescent fusion proteins facilitated the application of advanced super-resolution optical microscopy toward the visualization of the initiation of packaging. The formation of twin TerS ring complexes, each expected to be ~15 nm in diameter, supports a double protein ring–DNA synapsis model for the control of packaging initiation, a model that may help explain the variety of ring structures reported among *pac* site phages. The examination of the dynamics of the T4 packaging motor at the single-molecule level in these studies demonstrates the value of state-of-the-art fluorescent tools for future studies of complex viral replication mechanisms.

## 1. Introduction

All tailed phages package their dsDNA into a DNA-empty procapsid (or prohead) toward the end of infection to ensure that the viral genome is protected, and, after cell lysis, able to be transferred to new host cells to initiate the infection process once again. This critical process is highly conserved not only among tailed phages, but also among other duplex DNA-containing viruses, such as herpes- and adenoviruses [1,2,3,4]. In all these viruses, genome packaging into the procapsid employs a multimeric viral ATP-based DNA translocation motor comprising the large terminase protein or TerL [5,6,7]. The terminase interacts with a portal ring structure, located within a single procapsid vertex, to drive the DNA through the central cavity in the portal into the capsid. Akin to other large molecular machines such as the replisome, this phage DNA packaging complex of portal-containing prohead, terminase, and substrate is sometimes referred to as the “packasome” [8,9]. Once the appropriate length of genetic material has been packaged, the terminase ends the packaging process by cleaving the DNA. To achieve these very different functions, terminase proteins are large proteins comprising an *N*-terminal ATPase domain and a *C*-terminal nuclease domain that are joined by a flexible linker region [10,11]. Remarkably, nucleic acid packaging by these ATP-driven TerL proteins results in the packaged DNAs being condensed to a comparable degree, ~500 mg/mL, within the capsid, despite the wide variations in capsid dimensions and genome lengths that exist between different viruses [12]. To achieve this level of DNA condensation, DNA packaging motors must produce high forces, and this expectation has been supported by studies on multiple phage packaging motors, demonstrating their high usage of ATP (~2 bp/ATP) and production of comparably high forces (~60 pN (piconewton)) and translocation rates to fill their proheads with a genome’s worth of DNA during development [13,14,15].

DNA packaging is a critical area of viral assembly study for multiple reasons, including its bearing on DNA dynamics and structure, its relationship to condensed genome structure, and its relevance to medical fields—not least its potential for gene therapy [16,17]. However, since to date only phage proheads have been able to be filled in vitro to form an infectious virion, the knowledge derived from in vitro phage packaging studies has acted as a valuable proxy for their eukaryotic counterparts. The knowledge gained from phage DNA packaging research can be translational, as exemplified by the development of anti-HCMV terminase nuclease inhibitor AIC246 [18]. Similarly, in vitro packaging of any DNA and protein together into phage proheads designed to be tissue-targeted can be exploited for eukaryotic gene transfer [17]. And the knowledge derived from in vitro phage packaging studies is relevant to the phage field that is growing significantly due to the interest in phage therapy [19,20].

Studies on the indispensable components of the packasome motor have revealed many structural and mechanistic insights into packaging in some of the most rigorously studied model double-stranded DNA phages viz. T4, P22, SPP1, Phi29, and more recently environmental phage isolates [21]. These include crystal structures of the proteins that directly interact with the DNA during packaging, portal, large and small terminase subunits, and the major capsid protein [14,22,23,24,25]. Cryo-EM reconstructions of multiple components of the packasome, as well as a wealth of analyses produced via genetic, biochemical, biophysical, and structural approaches, have established an array of characteristics including those that are only conserved within some types of phages. For example, it has long been known that the physical ends of the packaged genome produced after cleavage by TerL can vary considerably between phage types. These differences result from variations in how different phages replicate their genomes, and/or the specificity of their terminase proteins to cleave or recognize certain signals in the long concatemeric DNA which is the substrate for packaging. For instance, many phages related to the *E. coli* phages λ and HK97 and the mycobacteriophages L5 and D29 have relatively short, complementary single-stranded DNA overhangs at their genome termini (cohesive or *cos* ends), whereas some phages such as *E. coli* phage T7 and *Bacillus* phage SPO1 have genome ends with direct terminal repeats [26,27,28,29,30,31,32]. That both *cos* ends and terminal repeats are present in phages infective for phylogenetically diverse hosts highlights the complex ancestry of DNA packaging—and these examples do not even represent the full array of genome end types. In contrast to these phages that package “unit length” genomes, the *E. coli* myovirus T4 produces virions with varying genome sequence coordinates among them. This is because T4 genome packaging initiates at a terminal packaging sequence or *pac* site and continues until a “headful” amount of DNA has been packaged, at which point TerL performs a terminal cleavage that is not driven by sequence specificity [33].

Due to the diversity that exists between the genomes, virions, packaging components, and mechanisms of different phages, as well as the many techniques used to study DNA packaging, it is not surprising that over time multiple mechanisms to explain the translocation of DNA during packaging have been put forward. For instance, an early model proposed that the DNA molecular motor operated via an F1F0 ATPase-like rotary mechanism, with biophysical approaches first suggesting otherwise [34], but the portal is now known to be immobile in terms of rotation, but is still a critical component of a linear motor for which the translocation force is provided by TerL’s ATPase activity [35,36]. One motor mechanism proposed for phage T4 DNA packaging involves a portal DNA-driven grip-and-release mechanism in which conformational change due to portal interaction drives DNA into the prohead via a compression motor stroke [37]. Another model for the T4 packaging motor proposed that a conformational change in the terminase pentamer when located on the portal supplies the necessary translocation force for packaging [24]. Recent structural analyses of the HK97 packaging motor support the highly dynamic nature of the packaging process and that terminase subunits undergo contraction and relaxation during DNA translocation, and the linking of ATP hydrolysis to contraction drives the substrate into the prohead [38].

For herpesvirus, a sequential revolution model was proposed in which DNA translocation and DNA cleavage are produced by the rearrangement of domains of a hexameric terminase complex [2]. The latter model shares features with that of phage phi29 [39], whose genome packaging process differs from those of many other phage types by virtue of its utilization of a packaging RNA (pRNA) that is believed to replace a *C*-terminal TerL domain that is missing in phi29 but conserved in other phage types [40,41]. Despite the significant differences between them, the various proposed packaging models have one point of similarity between them; that is, conformational changes in the motor proteins drive the linear motor responsible for translocation.

Clearly there are still fundamental gaps in the understanding of the DNA packaging process which are challenging to fill due to the highly dynamic nature and speed by which packaging proceeds (e.g., DNA translocation rates of up to 2000 bp/s have been recorded [13]). As part of the ongoing quest to better understand this process, Black and Ray laboratories commenced a collaboration ~15 years ago with the goal of applying single-molecule fluorescence approaches to elucidate mechanisms of DNA packaging in the T4 phage. Below, we briefly introduce the main components of the T4 DNA packaging system and the background and theory behind single-molecule fluorescence-based analyses of T4 DNA packaging. These are followed by a discussion of the findings derived from those fluorescent studies on the T4 packaging motor.

## 2. Main Components of the T4 Packasome

Phage T4 DNA translocation is driven primarily by the interactions of two major components, the large terminase protein (gp17) attached to the prohead portal (gp20). A dodecamer of gp20 forms a turbine-like structure at a single procapsid vertex that is ~12 nm long and comprises four major domains—crown, wing, stem, and clip—causing its external diameter to vary from ~8 to 17 nm [42]. A central channel runs through the center of the portal which also varies in diameter, being slightly over 4 nm at it its widest point, which is where it opens into the interior of the capsid, and ~2.8 nm in diameter at its narrowest point, roughly midway along the channel [42]. It is through this channel that DNA is pumped into the interior of the procapsid, which is ~100 nm long and 75 nm wide. After packaging is completed, the portal has additional essential functions in virion formation, including that a neck- or collar-like structure is assembled below it so the contractile tail can be attached. In addition, upon infection of a new host, the DNA (and internal head proteins) is ejected through the central channel of the portal and tail into the host cell.

T4 packaging initiates at a packaging sequence or *pac* site in the DNA that requires the small terminase protein (TerS, gp16) for its recognition in the freshly replicated concatemeric DNA. TerS’s role involves engaging TerL’s nuclease activity to make the initial cut in the concatemeric substrate so packaging can commence. After a set amount of DNA is packaged, this “headful” of DNA is sensed via the filling of the capsid shell, which induces conformational changes in the portal. The docked TerL is induced to cleave the concatemer again and, still associated with the DNA, disassociate from the prohead. The terminase–DNA complex is then able to dock onto another DNA-empty procapsid, and the process repeats. In this manner, 171 kb of linear DNA representing ~103% of the T4 genome, or the headful amount, is packaged into each capsid [43]. The additional ~3% more than the full genome map length that is packaged is referred to as a terminal redundancy.

Importantly, T4 DNA packaging can be carried out with up to 100% efficiency in vitro with linear DNA and two highly purified components (TerL and DNA-empty proheads) [44]. This in vitro packaging system and the T4 genetic system represent a powerful combination to study DNA packaging. It was strategic to apply fluorescence-based single-molecule-based approaches to study these systems since they have the potential to establish actual motor dynamics by monitoring in real time the various participants in the packaging process. Such experimental approaches are important to move beyond the depiction of static motor components docked hypothetically to rigid B-form DNA. Below, we briefly introduce the theory behind those fluorescent technologies, then follow with a discussion of their applications to study the roles and interactions of essential T4 DNA packaging proteins.

## 3. Single-Molecule Fluorescence Techniques: Significance and Impact

Single-molecule fluorescence techniques viz. fluorescence correlation spectroscopy (FCS) and single-molecule detection (SMD) provide a robust and reliable methodology to monitor the dynamics of DNA packaging in real time [45,46,47,48,49,50]. Moreover, this technique being noninvasive provides significant advantages over the traditional DNase protection assay. The underlying principle behind the FCS assay is to keep track of the development in the apparent diffusion coefficient when free DNA labeled using a certain fluorophore is translocated into a prohead. In an in vitro T4 system, double-stranded DNAs from 20 basepairs (bp) up to a 170 kbp genome have been reported to be packaged [51]. The measurement of DNA packaging using FCS depends to a significant extent on the variation in diffusion coefficients of the substrate and the prohead. A subfemtoliter volume is impinged onto an avalanche photodiode using an appropriate pinhole which aids in monitoring the fluorescence signal from that desired volume itself and cuts off any unwanted background signal. The autocorrelation function for *N* number of fluorescent particles traversing a 3D confocal with radius *w*_0_ and half-axial height *z*_0_ is given in Equation (1):

(1) $$G_{D}\left(\tau \right)=\frac{1}{N}{\sum_{i=1}^{n}f_{i}[1+\frac{4D_{i}\tau }{w_{0}^{2}}]}^{-1}{\left[1+\frac{4D_{i}\tau }{z_{0}^{2}}\right]}^{-0.5}$$

where *τ* is the lag time, *N* is the number of molecules in the confocal volume, and *f_i_* is the fraction of the corresponding diffusion coefficients *D_i_* [47,49,50,52].

Fluorescence-based methodologies (FCS, Förster Resonance Energy Transfer (FRET), and single-molecule (sm) FRET) applied to small linear DNA substrates with precisely located fluorophore adducts are also applicable to understanding the T4 packaging complex. This novel application of FCS and single-molecule technologies originally led to fluorescence assays of packaging in real time [50,53,54,55,56]. Single-molecule fluorescence techniques have proved to be an efficient tool in understanding the nature of macromolecular interactions in biological models. The strength of the technique lies in the fact that no specific synchronization of a molecular population is required for understanding the tracking of the reaction kinetics. FRET is a distance-dependent nonradiative process of energy transfer from a donor molecule to an acceptor molecule via dipole–dipole coupling. This results in corresponding enhancement of the acceptor emission and a concomitant decrease in the donor emission without the acceptor being directly excited. The process of FRET is highly efficient if the donor and acceptor moieties are within the Förster radius (3–8 nm). Another important aspect is the inverse dependence of the energy transfer efficiency on the sixth power of the intermolecular separation between the donor and acceptor, making it even more sensitive for probing processes that involve a change in intermolecular separation.

(2) $$R_{DA}=R_{0}{\left[\frac{1-E_{FRET}}{E_{FRET}}\right]}^{1/6}$$

where $R_{DA}$ is the intermolecular distance between the donor and acceptor, *R*_0_ is the Förster radius, and $E_{FRET}$ is the efficiency of energy transfer. In FCS, fluorescence intensity fluctuations of a very small population of the ensemble help in extracting dynamics of the system under investigation. In one of such initial studies, the open and closed ends of a DNA hairpin were labeled covalently using a fluorophore and quencher. It was observed that when the conformation of the hairpin was closed, the fluorescence intensity dropped owing to proximity of the fluorophore and the quencher, and it was restored once the hairpin retained its open conformation [45]. Thus, conformational variations lead to a fluctuation in intensity of emission which in turn reveals vital insights into the dynamics of conformational changes. Fluorescence approaches, combined with biochemical assays, appeared to have a significant potential to establish actual motor dynamics since they are capable of demonstrating structural changes and interactions that occur during translocation to both DNA substrates and the accompanying motor proteins.

## 4. Application of FCS and FRET to Monitor T4 DNA Packaging Events In Vitro

The goals of the first study of in vitro T4 DNA packaging using FCS were to establish the applicability of the approach to interrogate the T4 packaging process, and if it was applicable, to determine what were important parameters to consider in future studies [50]. Since the monitoring of diffusion coefficients was anticipated to serve as an important parameter in understanding the translocation kinetics of the reaction, 100 bp was selected for the substrate length since it has a very differently calculated diffusion coefficient to that for T4 proheads (38.0 μm^2^/s and 4.4 μm^2^/s, respectively) [50]. An important refinement to the packaging system established in this work was the inclusion of high-molecular weight PEG 20,000 in the packaging buffer to increase the viscosity of the reaction mixture. This slowed the diffusion of the DNA in the packaging buffer compared with if it was in Tris buffer alone. The DNA correlation curve fitted to a single exponential yielded a diffusion constant of 38.1 µm^2^/s in Tris buffer, almost identical to the calculated one. However, in the reaction buffer, owing to heterogeneity of the environment and interaction of the DNA with the polymers, a single component was not enough for fitting the correlation curves.

The diffusion coefficient of the proheads was assessed using products of a longer packaging DNA in which entire translocation of the labeled DNA occurred into the prohead interior. To estimate the kinetics of DNA packaging, autocorrelation curves were generated at regular time intervals for 30 min. Addition of ATP to the reaction mixture containing proheads and an excess of DNA initiated the reaction, and reaction mixtures in which ATP was not supplemented served as vital negative controls. Monitoring of the DNAs under these conditions every 20 s revealed that the diffusibility of the DNA decreased from the bulk of the surrounding solution relative to the phage interior by almost an order of magnitude due to the impediment of free diffusion of the DNA once it was packaged within the capsid. This observation was manifested as an increase in G(0) value as the reaction proceeded, indicating a decrease in the number of fluorophores in the confocal volume owing to multiple DNAs having been packed per unit of the prohead [50].

**Figure 1:**
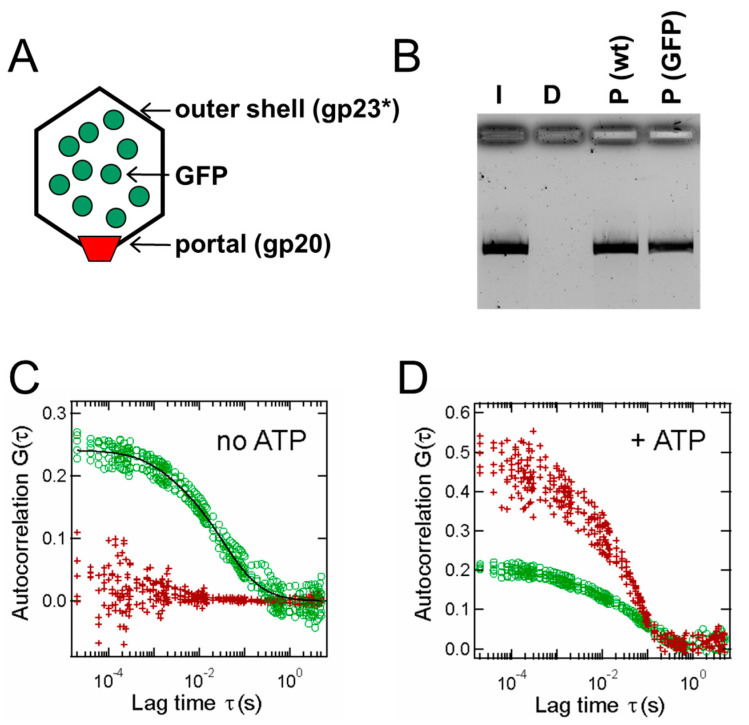
FRET-FCS of T4 DNA packaging in vitro. (**A**) Graphic illustrating that T4 capsids employed in the packaging assays had GFP as a foreign internal head protein via use of the T4 CTS system. (**B**) Example DNA gel of a nuclease protection assay. I indicates control input substrate DNA with no nuclease treatment, D indicates input DNA after treatment with DNase I, and P(wt) and P(GFP) indicate wild-type and GFP proheads, respectively, that were used for a nuclease packaging assay. (**C**,**D**) Overlaid FRET-FCS plots of five separate overnight packaging reactions without ATP (**C**) and with ATP (**D**) (GFP, green circles; TXR, red crosses). Adapted from reference [50].

Furthermore, in this study, a FRET-FCS assay was performed to confirm the proposition that DNA translocation had taken place into the prohead interior, i.e., to confirm that the observed changes in the diffusion coefficients were not merely artefacts produced by nonspecific interactions. The donor–acceptor pair used in this experiment was Texas Red/green fluorescent protein (GFP), with the Texas Red covalently bound to the DNA substrate and ~100 molecules of GFP located in the interior of each prohead having been packaged during prohead assembly by virtue of a capsid targeting sequence (CTS) fused to the GFP. In the Texas Red channel, the initially uncorrelated signal became highly correlated with the addition of ATP, which is due to FRET that can only happen when the donor and acceptor are within ~10 nm of one another (Figure 1) [50]. These data again demonstrated the efficacy of combining FCS and FRET methods in monitoring the translocation of the labeled DNA to the prohead interior.

This study not only established FCS as a robust and sophisticated technique to follow sequential DNA packaging in vitro but also revealed that if an initial packaging event did not go to completion, additional DNA–terminase complexes could bind to the portal and initiate additional packaging events, as deduced by the packaging of multiple (4–5) ~100 bp DNA fragments into a single phage prohead [50]. This latter finding was, and still is, remarkable for its implications regarding genome packaging in vivo as it supports the likelihood that in the event of an abortive packaging event (i.e., for some reason initially a headful of DNA was not packaged), additional DNA(s) could be packaged until the headful amount was reached. That DNA packaging may be reinitiated in vivo is intriguing, and is in stark contrast to other steps during phage infection that cannot be reinitiated after an aborted initial event. The capacity to reinitiate DNA packaging not simply once, but multiple times, might also represent the basis for the following: (1) a selective advantage for *pac* site phages over phages for whom the terminal DNA cleavage event at the end of packaging is dependent upon a sequence motif (e.g., *cos* phages), and (2) an important factor in both recombination and transduction events known to occur in T4-like phages [57,58,59,60].

## 5. DNA Substrate Structure Dictates Packaging Motor Efficiency

FCS studies, in conjunction with nuclease protection assays, were then performed to define which substrates the T4 packaging motor could, or could not, translocate into the prohead. Substrates varying in length, composition, and source (e.g., phage and plasmid DNAs), as well as many creative oligomer-based constructs, were assessed [37]. These studies confirmed that in vitro, and in the absence of the small terminase protein, the required nucleic acid substrate was dsDNA based on the inability of the packaging motor to translocate short RNA:DNA hybrids (20 bp) and longer 4 kb dsRNAs. Such substrate specificity is likely an important evolved “rule” to reduce the likelihood of the formation of defective particles during packaging as the cellular packaging environment is rich in both the RNA needed for late gene expression and concatemeric DNA. In addition, these studies demonstrated there were no apparent sequence requirements by the packaging motor as it was able to package various dsDNAs of many lengths (e.g., ~20 bp–48 kbp [37]). Conversely, investigations on the impact of substrate geometry on packaging revealed they could have a significant impact, some preventing packaging altogether, such as hairpin loops, whilst others modulated packaging in a contextual manner. For instance, short DNA mismatches (e.g., of 10 bp) could be packaged with reduced efficiency but longer mismatches producing larger D-loops were not packaged [37]. Similarly, DNAs with single-stranded overhangs up to ~12 bases on either 3′ or 5′ ends could be packaged, whereas those with longer single-strand overhangs (e.g., 20 or 40 bases) were packaged with less efficiency [37]. Substrate length was also an important parameter for the packaging of DNAs with single nicks. Longer 500 bp nicked substrates were packaged at the same efficiency as un-nicked controls, whereas shorter 100–200 bp length nicked substrates were packaged at reduced efficiency [37]. An FCS assay showed the latter result was caused by the shorter, nicked substrate not remaining stably bound by the packasome. These data highlighted the importance of having two unbroken strands for successful translocation, especially if there was no substantial leader sequence to the substrate that had already been packaged into the prohead to help it remain anchored to the motor in the event of translocation stalling structures. Importantly, this study demonstrated the exciting potential of using FCS, and facilitated the design of additional experiments to interrogate the relative locales of motor components and the dynamics of the packaging process.

## 6. Single-Molecule FCS Localization of the T4 DNA Ends within the Capsid

Building on the identification of substrate preferences by the T4 packasome, single-molecule FCS packaging assays were then employed to gain a new perspective on the location of the packaging termini within the capsid. It had been known for some time that during T4 packaging, the first mature genomic DNA end that was packaged into the procapsid was also the first end delivered into the host cell [51], but how the genome ends were coordinated within the capsid to facilitate such ordering was in no way clear. Based on its successful implementation to follow DNA packaging and detection of dye molecules within the prohead, it seemed feasible that FRET might lend itself to resolving this question via the labeling of the two ends of the same substrate DNA with different fluorescent dyes. The dyes employed to act as donor and acceptor molecules were Cy5 and Cy5.5, respectively, with the rationale behind their selection being the optimum FRET distances for that specific dye pair [55]. The termini of substrate DNAs of two lengths (5 kbp and 50 kbp) were labeled and the in vitro packaging of these dye pair DNAs was followed using FCS-FRET and single-molecule FRET (sm-FRET) and the Cy5-to-Cy5.5 FRET distances calculated using Equation (2) above.

**Figure 2:**
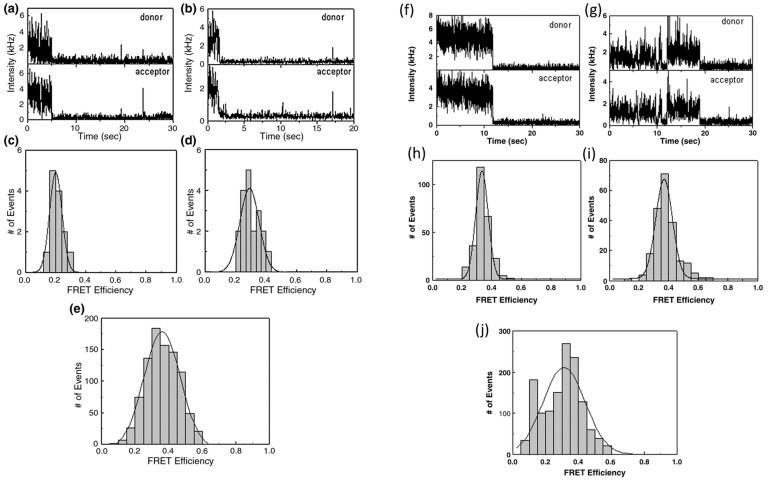
Single-molecule fluorescent intensity–time traces of immobilized packaged Cy5/Cy5.5-labeled 5 kbp DNA (**a**,**b**) and 50 kbp DNA (**f**,**g**) as seen in the donor and acceptor channels recorded simultaneously by two SPAD detectors. Corresponding FRET efficiencies from the double-dye-labeled packaged 5 kbp DNA (**c**,**d**) and 50 kbp DNA (**h**,**i**). (**e**,**j**) sm-FRET efficiency of packaged Cy5/Cy5.5-labeled 5 kbp DNA and 50 kbp DNA, respectively. Adapted from reference [55].

Notably, the experimentally derived FRET distances for the two dyes using FCS and SMD showed almost similar results whether the packaged dye-labeled DNA was 5 kbp or 50 kbp DNA in length: 9.3 nm and 8.6 nm, respectively (Figure 2). Since the FRET histogram distribution was very tight, these data strongly support the interpretation that the ends of both lengths of packaged DNA were held at almost the same distances within the capsid. Based on these results, it seems likely the localization of the DNA ends after in vivo packaging also holds true to these in vitro observations. The resultant packaging of the full-length 170 kbp genome as a loop with one end tethered to the top of the portal may be an important mechanism to prevent knotting of the genome within the capsid, the latter being a structure that would effectively make a particle nonviable due to the roadblock it would create for DNA ejection during infection.

## 7. Portal Control of the Prohead Expansion Necessary for Genome Packaging

The portal is clearly a critical component of the packasome, but it also has additional functions during virion assembly, as noted above, and a better understanding of the T4 portal’s role prior to packaging was needed to optimize in vitro packaging studies. T4 head assembly initiates with the formation of an oval-shaped protein core above the portal structure, which is anchored to the inner *E. coli* membrane. The outer capsid protein gp23 assembles around this core. Proteolytic cleavage by the T4 prohead protease then removes propeptides from head proteins, including gp23, and cleaves core proteins to small peptides that exit the capsid. The conformational changes in the prohead resulting from this proteolytic maturation result in the prohead being released from the *E. coli* inner membrane to which it had been bound via the portal, and its outer shell expanding, steps that are clearly critical for DNA packaging to occur. At this stage, two prohead species, empty small proheads (esps) and empty large proheads (elps), were known to form in vivo, and as their names imply both are DNA-free. Both esps and elps comprise the same proteins, but they vary in dimensions, structure, stability, and charge, enabling them to be separated by ion exchange chromatography [61,62,63].

Although esps matured to elps in terms of capsid shell expansion, the role of these two prohead species in terms of DNA packaging, and therefore the suitability of each species for in vitro packaging studies, was unclear. To address those questions, the efficiency of DNA packaging was assessed for wild-type (WT) elps and esps and also for proheads in which the *C*-terminus of the portal proteins was fused with GFP, causing the GFP to locate in the prohead interior [52]. The latter were generated by expressing the gp20-GFP in trans from a plasmid during nonpermissive infections using several mutants lacking a WT portal gene and other relevant genes (e.g., *16-* and *17-*). In contrast to WT infections in which types of proheads are observed, repeated infections to generate proheads containing gp20-GFP proheads resulted only in the incorporation of the fusion protein into esps, suggesting gp20-GFP portals in some way hindered the esps to elps transition [52]. However, when a mutant with active terminase genes and only a single mutation (*20-*) was used to create proheads with gp20-GFP, a similar ratio of gp20-GFP esps and elps as in WT infections was produced, supporting that an active terminase protein was in some way linked to prohead expansion and in the absence of DNA packaging the proheads were effectively locked into the esp state [52].

**Figure 3:**
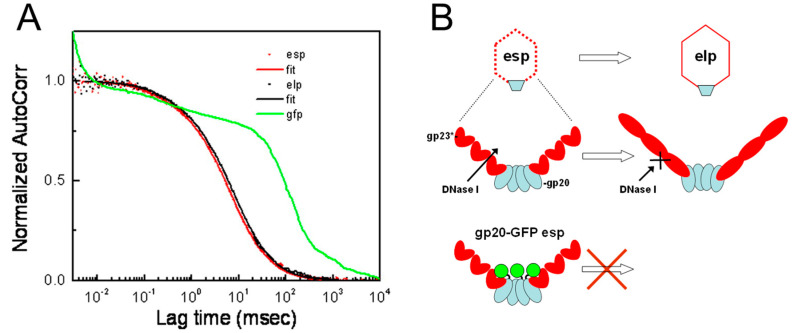
FCS analyses of T4 DNA packaging support the portal having a central role in expansion of the major capsid protein lattice. (**A**) FCS analysis of packaging into esps (red), elps (black), and gp20-GFP (green) proheads showing that capsid expansion of proheads is not necessary for DNA packaging. Packaging assays included 100 bp Rh-tagged DNA, purified gp17, and purified proheads in varying forms, and were incubated in a buffer containing ATP. The autocorrelation (each normalized to unity) is presented. Data obtained with esp or elp proheads are shown as points, and curves fitted with a two species diffusion model are superimposed as lines. The autocorrelation curve calculated from analysis of packaging assays performed with the gp20–GFP proheads is also shown. (**B**) Scheme showing the importance of capsid expansion for the protection of packages from external nuclease, conclusions derived from both FCS and nuclease protection assays. Reproduced from [52].

Nuclease protection packaging assays revealed that WT elps packaged short DNAs very efficiently, more so than longer DNAs, whereas WT esps and gp20-GFP-containing esps and elps had much lower packaging efficiencies for short substrates [52]. But gp20-GFP-containing esps and elps were able to package longer substrates more efficiently. In contrast to the nuclease assays, FCS assessment of packaging assays using a short 100 bp rhodamine substrate and gp20-GFP esps or elps revealed that most of the input DNA was sequestered into esps or elps proheads after a 60 min packaging reaction (Figure 3). That is, in contrast to the nuclease assay results, the FCS data implied that both the esps and gp20-FP elps were as active as the elps in packaging short DNA molecules [52]. Together these data clearly supported that the esp to elp expansion was blocked until a critical-length DNA was packaged in gp20-GFP esps and also that that transformation in elps enables their DNA to be protected from DNase. Not only were these experiments relevant in the context of understanding the portal control of T4 capsid lattice expansion and its capacity to package full-length genomes, they were crucial for the success of following packaging studies, by establishing which prohead population was the most appropriate to employ.

## 8. Packaging-Induced Conformational Change in Substrate DNA by Motor Proteins

The previous studies characterizing the impact of substrate nicks, gaps, or branches on packaging efficiency in vitro were significant as they aligned with earlier genetic work showing those structures were present in the in vivo substrate based on observations that deficiencies in ligases (T4 or *E.* coli), T4 endonuclease VII (endo VII), or topoisomerase II all negatively impacted the formation of DNA-full heads (e.g., [64,65]). Importantly, analogous to such genetic studies that can provide deep insight into gene function via the comparison of permissive and nonpermissive conditions, the determination that T4 packaging could be controlled in vitro via substrate design paved the way for groundbreaking fluorescence-based studies to interrogate a proposed torsional compression translocation model.

The first evidence of substrate compression during T4 DNA packaging was obtained via stalled packaging assays that employed dye-labeled Y-DNA substrates that had a dye molecule located at the branch point [56]. Initially, a suitable length for such a stalled substrate was established using Texas Red dye (Tx)-labeled Y-DNAs in assays with procapsids containing either a wild-type portal or a portal in which four to six of the portal monomers were replaced with gp20-GFP fusion proteins. In those assays, as observed previously, substrate length was an important determinant for the packaging of nonlinear dsDNA. A short 90 bp TxY-DNA was not packaged, as indicated by its digestion in a nuclease packaging assay, whereas when a 2.7 kb leader sequence was ligated to the stem of the TxY-DNA, most of the stem was protected from the nuclease assay, inferring it was packaged [56]. Similarly, no FRET was detected between the short TxY-DNA and portal-bound GFP, but FRET was recorded between the donor pair when the 2.7 kb TxY-DNA substrate was used in the packaging assay. The diffusion coefficient of the portal-anchored 2.7 kb TxY-DNA was found to be comparable to previous experiments, and FRET measurements facilitated an estimation of ~5.8 nm for the donor–acceptor pair distance, assuming a 4 nm Förster distance for a GFP-Tx pairing. These results aligned with the leader sequence having been packaged but stalled at the Y-junction [56].

**Figure 4:**
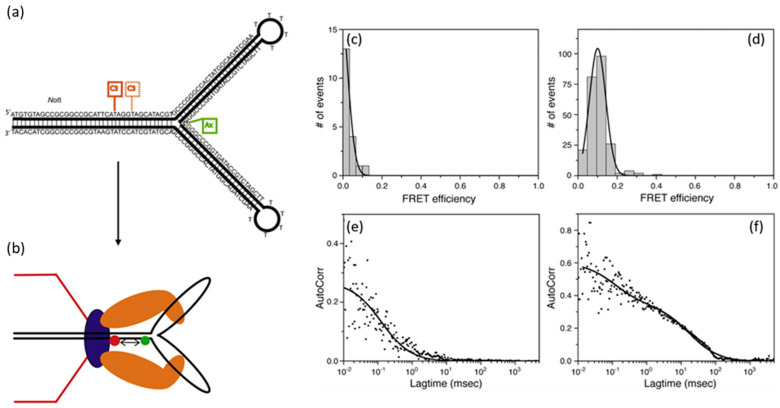
(**a**) Depiction of structure and construction of the energy transfer dye pair 2.7 kb AxCyY-DNAs with 10 bp and 14 bp spacing and (**b**) representation of the base of a normal procapsid portal-bound terminase (portal, blue; terminase, brown) with an anchored 2.7 kb AxCyY-DNA to measure packaging-associated FRET change. Panels (**c**–**f**) TxY-DNA stalls in proximity to GFP-containing portal by FRET. (**c**) No FRET of TxY-DNA in absence of leader and packaging; (**d**) FRET showing GFP to Tx when 2.7 kb TxY-DNA is packaged. Autocorrelation plots of Tx-Red channel are shown in panels (**e**) and (**f**). Adapted from reference [56].

To then investigate the orientation of the stalled Y substrate, assays were conducted using 92 bp Y-DNAs labeled with a dye pair: a donor Alexa488 (Ax) at the Y-junction and an acceptor Cy3 (Cy) located 10 or 14 bp down the stem from the junction (Figure 4) [56]. As in the TxY-DNA experiment, the short AxCy-Y-DNA was also not able to be packaged, but after ligation with a 2.7 kb dsDNA stem, a nuclease assay indicated a portion of the longer Y-DNA had been packaged and correlation spectroscopy showed the packaging mixture to now have a diffusion coefficient that was procapsid-like [56]. To test the expectation that the motor was stalled with the branch of the Y-DNA unable to be packaged, the samples were treated with nuclease. This resulted in the fluorescence diffusion coefficient changing to one indicative of high mobility, showing that a dye-labeled residue was indeed not in a protected location (i.e., within the prohead) and only the stem had been packaged. The FRET efficiencies of the stalled 10 bp or 14 bp spaced AxCy-Y-DNA were found to have two components, a ~20% and 70% efficiency transfer for the 10 bp spacing, versus 15% and 55% for the 14 bp spacing. Based on comparison with measurements of packaging controls to which no terminase or no proheads had been added, the higher energy component measured for each AxCy-Y-DNA had to derive from them having been packaged but stalling the motor at their branches. Extrapolation of the differences in those efficiency transfer values revealed changes in the Y-stem inter-dye spacings of ~24% and 22% for the 10 bp and 14 bp dye pairs, respectively [56]. Those changes logically derived from the compression of rigid B-form DNA by a linear force while it was constrained by the packaging motor within the narrow portal channel, lending support for a torsional compression model for packaging via substrate compression by the terminase and a release step by the portal.

## 9. Spatial Assessment of Stalled and Released Substrates during Packaging

Although other motor proteins such as helicases and RNA polymerase were known to infer structural changes on their substrate, the possibility that DNA was actually compressed by the packaging motor was surprising as it opposed the idea that the force from a linear motor would result in the stretching of DNA. To further interrogate this substrate “crunching” phenomenon, a series of elegantly designed fluorescence-based experiments were undertaken that employed remarkably different substrates, including additional stalled Y-DNAs, linear DNAs labeled with intercalating dyes, and short heteroduplex DNAs. Notably, the results of all experiments aligned with a transient compression of the substrate DNA that was accompanied by proportional relative changes in the main motor proteins.

**Figure 5:**
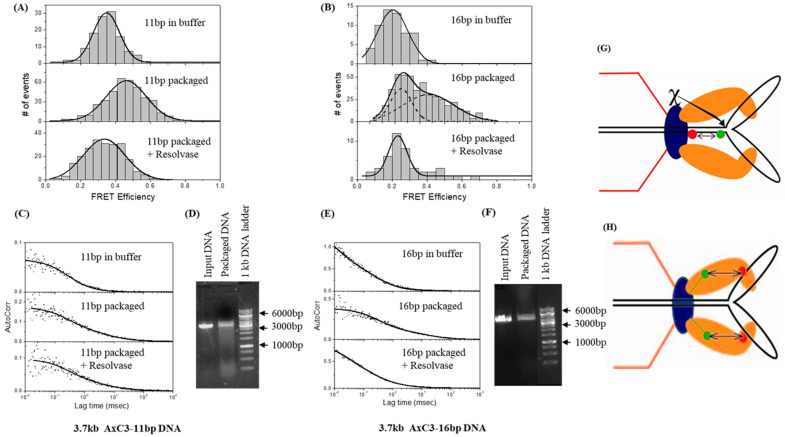
T4 Endo VII resolvase affects the compression of 11- and 16-bp Y-DNAs. (**A**,**B**) FRET-FCS measurements of 3.7-kb AxC3Y-DNAs with 11-bp (**A**) and 16-bp (**B**) inter-dye distance DNAs in buffer and in packaging mix before and after resolvase addition (1 µL to 16 µL packaging mixture) were carried out at room temperature. (**C**,**E**) Autocorrelation plots to measure diffusion of the unpackaged, packaged with, and without resolvase 11-bp (**C**) and 16-bp (**E**) 3.7-kb AxC3Y-DNAs. (**D**,**F**) Nuclease assays were carried out following FCS-FRET measurements confirming the packaging of the 11-bp (**D**) and 16-bp (**F**) 3.7-kbAxC3Y-DNAs. The markers are shown with arrows. (**G**) Schematic of FRET between different dyes within the DNA and site of action of gp49. Portal-bound Endo VII resolvase action releases the compression from the Y-DNA substrates. (**H**) Resolvase release of trapped Y-DNAs is also correlated with increased distance (~0.6 nm) between the terminase and portal as shown by changes in FRET between the labeled terminase and portal during packaging. Adapted from reference [66].

In an additional study of stalled Y-substrate DNAs, the addition of the T4 gp49 Endo VII resolvase was assessed to determine its impact. Endo VII resolvase is an essential Holliday junction resolvase whose role in late DNA replication is well studied, but it is also known to be closely associated with the packaging proteins in vivo [8]. Initially, a 3.7 kb Y-DNA substrate labeled with Texas Red at the Y-junction was packaged into gp20-GFP proheads to ensure that comparable results could be obtained with this slightly longer substrate. Nuclease protection assays confirmed that most of the DNA had been internalized into the prohead and FRET-FCS of the stalled complex again estimated ~5 nm distance between the donor and acceptor dyes. After confirmation that the Y-stem of the 3.7 kb Y-DNA was cleaved by the gp49 resolvase using gel mobility assays, its impact on stalled Y-DNA packaging mixes was assessed. For those experiments, the stems of the Y-DNAs were labeled with a Cy3 acceptor dye in the stem either 11 or 16 bp apart from an Ax donor dye located on the complementary strand. After packaging, the calculated distances for the donor-to-acceptor dye pairs differed for each substrate, being 0.6 nm for the 11 bp AxC3Y-DNA and 1.6 nm for the 16 bp AxC3Y-DNA (Figure 5), which equate to ~9% and 18% changes in the inter-dye spacing, respectively [66]. That is, there was an average DNA compression of 10–20% that was associated with packaging. After addition of purified resolvase to packaging reactions with each substrate, there was a decrease in the FRET efficiency reflecting an increased distance (0.6 nm) between the dye pairs, consistent with the linearization of the Y-DNAs and completion of their packaging (Figure 5).

## 10. Spatial Assessment of Motor Components during Packaging

To investigate the interactions between both the terminase and substrate and the terminase and portal, various dye-labeled motor proteins were created. Initially, the spatial interactions of two TerLs were assessed, one labeled on its *N*-terminus, the other on its *C*-terminus with ReAsH-EDT2 dye (NT-ReAsH and CT-ReAsH, respectively), both of which were shown to be fully active [66]. The packaging reactions with these terminases contained a 3.7 kb Y-DNA labeled with a single Alexa 488 dye and WT proheads. The FRET efficiency values obtained differed for the two terminases, with 35% recorded for the CT-ReAsH terminase, corresponding to a shorter donor-to-acceptor dye distance (6.8 nm) than the 20% FRET efficiency value obtained for the NT-ReAsH terminase, which was equated to a 7.8 nm donor-to-acceptor dye distance [66]. Terminase-to-portal interactions were also assessed for the two ReAsH terminases in packaging reactions with GFP-gp20 proheads and unlabeled substrates (linearized plasmid DNA or Y-substrate). A FRET value of 20% was observed between the NT-ReAsH terminase and GFP-gp20 portals which equated to a donor-to-acceptor distance of 6.9 nm. Intriguingly, the FRET efficiency values for the CT-ReAsH terminase varied considerably depending upon the substrate employed, with FRET values of 60% versus 45% observed for 3.7-kb Y-DNA and linear DNA, respectively. These values corresponded to donor-to-acceptor distances of 5.1 nm and 5.7 nm between the *C*-terminus of terminase and portal for stalled versus un-stalled substrates, respectively [66]. Addition of the gp49 resolvase to the packaging mix employing the Y-DNA resulted in a decrease in the higher FRET efficiency value to 45%, consistent with the substrate having been linearized and confirming the CT-ReAsH and portal were more closely located when the packaging motor was stalled. Together, the correlated protein and DNA conformational changes observed in this study were strongly indicative of DNA packaging being a highly dynamic process, and that TerL underwent significant conformational changes during DNA translocation. The spatial changes observed could also be interpreted as reflecting relaxed versus tense motor states in arrested (Y-DNA) versus translocating states (linear DNA).

**Figure 6:**
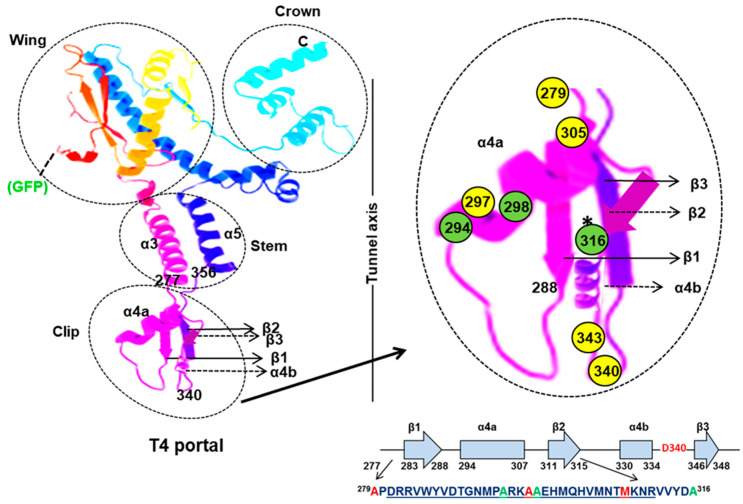
Relative locations of T4 portal substitution mutations employed for FRET of portal–terminase interactions. Left: tertiary structure prediction of the T4 portal using the Phyre2 with the crown, wing, stem, and clip regions indicated. Right: T4 portal clip region with amino acids that could not be substituted by cysteine (279, 297 and 305) indicated with yellow, and other amino acids (340, 343) that were substituted with cysteine indicated in green. FRET transfer between CT-ReAsH terminase and Alexa 488-labeled proheads was observed with the A316C residue modification. The position of the *N*-terminal GFP in the predicted T4 portal is shown. Adapted from reference [68].

The determination that the *C*-terminus of TerL was in a closer locale to the portal than its *N*-terminus by FRET seemingly conflicted with results from structural investigations [24], leading to additional investigations to assess the relative spatial orientation of the packaging motor components. The following study sought to replace the GFP fluorophore fused to the *C*-terminus of the portal protein (likely placing it exterior to the wing domain of the portal protein) used in the previous study with a maleimide dye-labeled cysteine residue in a region of the portal protein expected to be located close to the terminase protein during translocation. The region selected to target was residues D279-A316 of gp20, as that region was indicated as a terminase interaction region by earlier genetic, biochemical, and immunology studies [67]. Bioinformatic analyses and comparisons with the SPP1 portal structure supported those earlier conclusions indicating that that region was located in the clip region at the base of the portal structure (Figure 6). Of six residues targeted for mutation to cysteine in that region, only three poorly conserved residues actually allowed the substitution mutation; that is, the codon for the mutation was able to recombine into the mutant used for prohead preparations and that mutant was still viable under permissive conditions (Figure 6). One of the cysteine portal clip mutations was successfully labeled using Alexa 488 maleimide dye and shown to be packaging-competent. FCS-FRET employing those maleimide Alexa488 dye-labeled A316C proheads, gp17 CT-ReAsH, resulted in a FRET value of more than 20% and an FCS diffusion coefficient comparable to that from previous measurements of the prohead (~2 μm^2^/s). That FRET coefficient equated to a donor-to-acceptor distance of ~7.5 nm, a distance that strongly supported the previous findings in which the *C*-terminal end of the terminase was indicated to be closer to the GFP-labeled portal (gp20) than the *N*-terminal end of the terminase.

## 11. Insight into the Role of TerL Using Intercalating Dye-Labeled and Heteroduplex Substrates

Intercalating acridine dyes are well known to prevent the formation of viable particles in many phages, resulting in the accumulation of DNA-empty heads leading to the inference that these dyes in some way impacted the DNA packaging motor. Supporting these, resistant mutants in T4 have been isolated that have mutations in the gp17 called *ac* and *q*, as they confer resistance to acridine and quinacrine, respectively. Knowledge of both these mutations and the structural impact of intercalating dyes on DNA led to a creative study to better understand permitted substrate permutations during DNA packaging [54].

**Figure 7:**
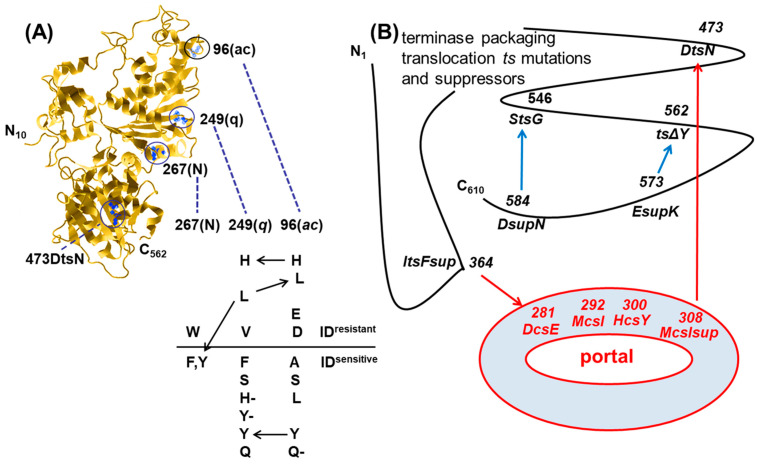
T4 terminase intercalating dye-resistant mutations and terminase–portal interacting translocation functions. (**A**) ac96, q249, and N267 sites linearly align with each other and with portal clip interacting residue 473 (circled in the gp17 monomer structure) and show complex interdependencies in ID resistance and terminase function. Various interdependent (shown by arrows) amino acid substitutions at residues 96(ac), 249(q), and 267(N) in the terminase leading to either ID resistance (above line), sensitivity (below line), or loss of terminase function (–) are shown together with the 3D structure. (**B**) Packaging translocation temperature-sensitive (*ts*) and cold-sensitive (*cs*) mutations and intra- and intergenic suppressors of the packaging motor proteins (*ts* mutants have gene variants for which the standard growth temperature is permissive but elevated temperatures are nonpermissive, and conversely, a higher temperature is permissive for the growth of *cs* mutants but a lower temperature is non-permissive). Terminase *ts* mutations are marked in black, and portal *cs* mutations are marked in red. Intragenic (18) suppressors are shown with blue arrows, and intergenic (–21) suppressors are shown with red arrows. Only the portal clip region and translocation channel are shown. Reproduced from reference [68].

The intercalating dye YOYO-1 was selected for study due to its extremely high affinity for DNA, and it was shown to inhibit wild-type TerL gp17 packaging both in vivo and in vitro. Sequencing of the *ac* and *q* mutant terminase genes revealed that the mutations *ac*-A96D and *q*-F249V (Figure 7) were responsible for their phenotypes and that DNA packaging employing such mutants in the presence of intercalating dyes resulted in the removal of those dyes. Even in the absence of proheads and ATP, ~18–35% of the YOYO-1 bound to DNA was shown to be removed by the mutant terminases. However, in the presence of all packaging components the results were significantly different, with all detectable YOYO-1 being eliminated from packaged substrates ranging in length from 70 to 280 bp. The removal of intercalating dyes was surprising in that the earlier studies had demonstrated that DNAs covalently linked to them could be packaged; however, the discovery of this removal of intercalating dyes by the packaging motor effectively supported that it functioned via a linear DNA grip-and-release motor mechanism transiently compresses B-form DNA during translocation.

**Figure 8:**
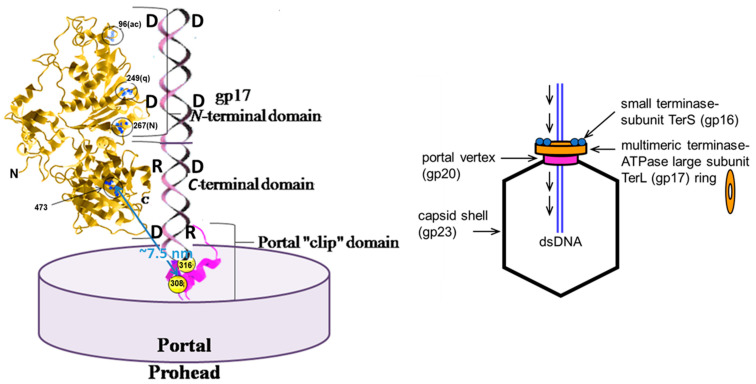
Phage T4 prohead portal gp20 binding to TerL from the C-terminal. Two duplex nucleic acid segments are depicted as being translocated by the TerL ATP-driven motor, where the top segment is pushing the bottom segment through the portal tunnel into the prohead. TerL intercalating *ac* and *q* dye-resistant mutations at residues 96, 249 are circled; mutations at residue 267 are closely coordinated with mutations at the *ac* and *q* sites; the circled residue 473 *ts* DNA translocation mutation is suppressed by portal *cs* mutation (*cs,* cold-sensitive) clip domain residue 308 (blue arrow). A FRET determined distance of ca. 7.5 nm between portal clip domain Alexa488 maleimide dye-labeled residue 316 and the terminase C-terminal ReAsH dye is found in this study (blue arrow). Structures are not to scale, nor are the numbers of terminase or portal monomers represented in the multimeric structure. Inset shows a scheme of conserved bacteriophage two-subunit terminase prohead packaging. Adapted from reference [69].

Additional support for the transient compression of the substrate from B-form to an almost A-form DNA structure by the T4 motor was built on the earlier findings—noted above—in which the T4 motor was shown to be able to package multiple short substrates into the prohead in vivo. In this study, it was demonstrated using FCS-FRET that multiple copies of fluorescent dsDNAs can be packaged in a single prohead [69]. The packaging efficiency remained almost similar for both 20–30 bp DNAs and 40–100 bp DNAs but shorter-length (<15 bp) DNAs could not be packaged, presumably because of their being too short to completely engage the TerL translocation chamber. The efficient packaging of the 20–30 bp DNAs pointed toward the substrate having to be pushed by the ~100 Å long packaging motor rather than being pulled. The fact that 20 bp DNA:RNA heteroduplexes were actually packaged even more efficiently by gp17 TerL in vitro than the longer heteroduplexes provides credence to the expectation that in the packaging of short duplex nucleic acids a single molecule promotes translocation by helping another molecule by virtue of pushing (Figure 8). This report provided substantial evidence in favor of a B-form to A-form spring-like packaging motor that has the potential to recognize and efficiently package A-form D:R oligonucleotides.

## 12. The Elusive Role of TerS in the Initiation of DNA Packaging

The small terminase protein, TerS, has represented a tantalizing problem for the packaging field, caused in part by its poor evolutionary and structural conservation [70], completely setting it apart from the highly conserved TerL. The functional mystery of TerS in part results from the various numbers of monomer subunits (8–12) that have been reported for the crystal ring structures formed by different phage TerS proteins. Additionally, the peptide components of this small protein have been able to be shuffled without a loss of function. Such data have proved challenging to interpret and link the structural properties of TerS with its function, with one general rule that appears to hold for all *pac* site phages: TerS forms a ring structure to initiate packaging at specific sites in concatemeric DNA.

**Figure 9:**
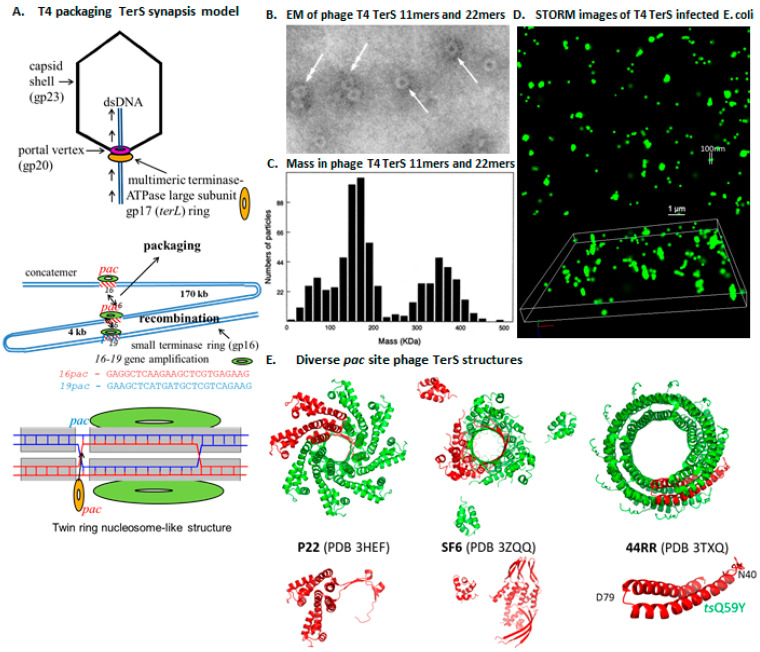
Conserved bacteriophage two-subunit terminase prohead packaging; (**A**) large terminase-ATPase subunit TerL (T4 gp17) only packages linear DNAs in vitro; small terminase subunit TerS (T4 gp16) required in vivo for concatemer packaging; a synapsis model proposes sequence-specific terminase gene amplifications that require TerS (gp16) apposition of two *pac* sites under genetic selection; and a two-ring TerS Holliday junction signal to initiate handoff to TerL for DNA cutting and packaging. (**B**) Negatively stained micrograph of T4 TerS EM single- and double-ring structures. (**C**) Mass determination of T4 TerS protein; (**D**) 2D and 3D super-resolution microscopy (STORM) images of YOYO-1 stained T4 TerS mCherry-infected *E. coli*. (**E**) Diverse *pac* site phage TerS structures (adapted from [77] and references therein).

Genetic analyses initially indicated T4 gp16 to be essential and since infections under nonpermissive conditions produced empty proheads that accumulated in the cell, clearly gp16 was in some way involved with the production of mature DNA-full heads. Later studies confirmed T4 TerS as being required for DNA packaging in vivo, and also in vitro when the substrate is highly branched concatemeric genomic DNA [71]. Conversely, gp16 was found to not be required and to be actually inhibitory in vitro for the packaging of linear DNA [60,72]. Early evidence for the T4 *pac* site was the preferential binding of TerS to a GC-rich sequence at the 3′ end of the gp16 gene [60,73]. In support of its *pac* site assignment, this sequence was also able to promote the transduction of plasmid and phage DNA [60]. Remarkably, the gp16 *pac* site was shown to participate in recombination with a homologous sequence in gp19 to produce gene amplifications of the intervening region in vivo when there was a selective force for increased synthesis of TerL [74]. Importantly, these gene amplifications did not occur after mutation of the *pac* sequence. This genetic evidence strongly supported that the function of gp16 and its *pac* site was in some way linked to homologous recombination, helping nucleate a model to explain the role of gp16 in DNA packaging [9]. Also integral to the development of that model was that purified recombinant gp16 (i.e., DNA-free) was shown to oligomerize single- and double-ring structures via electron microscopy (Figure 9) that had stoichiometries of 11- and 22-mers, respectively, that were determined via mass spectrometry [73,75]. These data resulted in a double-ring synapsis model to explain the role of gp16, which proposed that rings of gp16 acted akin to “lock-washers” binding DNA in a manner that enabled DNA to be bound, “handed off” to TerL for the initiation of DNA packaging, and also to promote synapse formation between homologous sequences (Figure 9). After the imaging of the T4 gp16 rings, crystal studies by the Rao and Rossman laboratories showed the formation of TerS 11-mer and 12-mer rings for gp16 of phage 44RR [73] (Figure 9), which is a T4-like phage [76]. They also undertook mutational analyses of T4 gp16, showing it to have an overall domain structure analogous to the TerS proteins of other phages: an *N*-terminal DNA-binding domain, a central domain responsible for ring oligomerization, and a *C*-terminal large terminase-binding domain [76].

To further interrogate the function of TerS using fluorescence approaches, the genes for eGFP and mCherry were each fused to the *C*-terminus of the gp16 gene and cloned in an expression vector [77]. Remarkably, induction of each TerS-fusion protein in cells infected with a gp16 amber mutant complemented the growth of the mutant at levels comparable to a recombinant gp16-no fusion control [77]. Superose column purification of the TerS-fusion proteins produced highly fluorescent >670 kDa multimeric ring complexes whose variable multimer sizes hindered structural analyses [77]. Mixtures of the purified TerS-mCherry and TerS-eGFP showed no FRET transfer between the donor and acceptor fluorophores; however, after renaturation using urea, ~50% FRET was observed [77]. The diffusion coefficient of this renatured TerS-fusion protein ring mixture was 17 ± 3 μm^2^/s compared with ~280, ~50, and 4.4 μm^2^/s for rhodamine, native GFP, and T4 proheads, respectively, which aligned with the molecular mass of the ring estimated via Superose purification [77]. Building on these properties of the TerS-fusion proteins, the genes for each were recombined into the T4 genome and the proteins were again found to be functional via the production of a “pseudo wild-type” phage, although in this instance they were expressed from their native promoter. Infections at high MOI by both TerS-GFP and TerS-mCherry phages were found to have a ~50% FRET transfer value similar to that of the protein average with only ring-like structures [77].

Building on these innovative experiments, two additional fluorescence-based experiments support that the formation of multiring structures is central to the function of the TerS [77]. One involved the introduction of a temperature-sensitive mutation into the central domain of the gp16 gene in the TerS-mCherry protein construct. Mixing and renaturing of this *ts* TerS-mCherry with the wild-type TerS-eGFP protein blocked FRET at high, but not low temperatures, presumably because the formation of ring-like oligomers was not feasible at high temperatures and impacted TerS function. The second experiment employed STORM and PALM super-resolution optical microscopy to assess the impact on TerS ring formation during T4 infection in the presence or absence of functional TerL (e.g., Figure 9). To make these comparisons feasible, additional plasmid and phage constructs were employed and exploited the photoactivatable version of mCherry (PAmCherry) fused to the *C*-terminus of TerS as well as a TerL with a known acridine resistance mutation and the use of the intercalating dye YOYO-1. These experiments showed there to be enhanced fluorescent intensity derived from photoactivated TerS proteins when there was no functional TerL, again supporting that TerS forms two, possibly more, closely associated rings or ring-like structures in vivo which are an integral component of the TerS ring synapsis model.

## 13. Conclusions and Future Directions

The implementation of FCS, FRET, and smFRET in the context of the T4 packaging motor resulted in the first fluorescence assay of DNA packaging in real time [50]. Subsequent studies established the potency of creatively implemented single-molecule detection techniques to interrogate the T4 DNA packaging process and generate novel insights into packasome function and dynamics. Various precisely located fluorophore adducts were used to demonstrate that while the TerL motor can package linear dsDNA of literally any sequence, more elaborate rules govern TerL’s ability to package substrates with more complex geometries. These rules align with the biological function of the motor late in infection, particularly the branched substrates that exist in newly replicated T4 DNA. Branched Y-substrates were shown in vitro by FCS-FRET to stall packaging and compress motor components. However, upon addition of the in vivo solution to branched structures, the Endo VII resolvase, FCS-FRET recordings indicated a release of the substrate compression concomitant with the recommencement of packaging. These findings aligned with a packaging model in which the substrate DNA undergoes torsional compression during translocation resulting from conformational changes in the packaging motor. The results from additional single-molecule fluorescence-based analyses also favored compression of the DNA during packaging. These included FRET analyses of TerL proteins with *N*- or *C*-termini labeled with ReAsh dyes enabling their localization to dye-labeled Y-DNAs and portal proteins, as well as TerL proteins with acridine resistance mutations that permitted them to package intercalating dyes known to normally inhibit packaging, such as YOYO-1. Additionally, other single-molecule fluorescent studies of dye-labeled portal and terminase proteins with temperature (*ts*) or amber (*am*) mutations also supported the transient spring-like B-form to A-form-like compression of DNA during translocation and that this compression correlated to movement of the TerL motor whose *C*-terminal domain was closer (docked) to the prohead portal than its *N*-terminal domain.

Novel analyses of fluorescent dye-labeled TerS proteins provided evidence in support of a proposed twin ring *pac* synapsis model to explain the initiation of T4 packaging. Together these studies have unequivocally demonstrated that fluorescence-based approaches can document structural changes in both the DNA substrate and the T4 motor proteins responsible for substrate translocation with numbers of findings providing insight into decades-old mechanistic questions.

The power of single-molecule fluorescence techniques lies in their ability to establish actual motor dynamics, and this expectation has been further exemplified in recent studies by other groups, such as one which redefined the ATPase Walker B motif in T4 gp17 [78]. That study employed total internal reflection fluorescence (TIRF) microscopy to study terminases in which all nine cysteine residues were mutated and then single-cysteine moieties introduced at defined positions with the aim of monitoring the translocation of the mutant motors [78]. Similarly, another multipronged approach study employing TIRF revealed that the T4 motor can accommodate nonfunctional gp17 subunits and still function, albeit with reduced packaging efficiency [79]. This led to the conclusion that the T4 motor can resume DNA translocation by skipping one subunit, i.e., T4 does not require the synchronization between all the terminase subunits as observed in other phage types [79].

Single-molecule fluorescence tools clearly hold significant potential for additional studies designed to derive additional mechanistic insights into T4 DNA packaging, such as into defining how the small terminase subunit acts to initiate packaging at *pac* sites and how its small ring structure relates to function, as well as into other infection steps of T4 and other tailed phages.

## Data Availability

Not applicable.
